# [Expression of Concern] Promoter methylation of death-associated protein kinase and its role in irradiation response in cervical cancer

**DOI:** 10.3892/or.2026.9125

**Published:** 2026-04-27

**Authors:** Rebecca Ching-Yu Leung, Stephanie Si Liu, Kelvin Yuen-Kwong Chan, Kar-Fai Tam, Kar-Loen Chan, Ling-Chui Wong, Hextan Yuen-Sheung Ngan

Oncol Rep 19: 1339–1345, 2008; DOI: 10.3892/or.19.5.1339

Following the publication of the above paper, and an Expression of concern statement (DOI: 10.3892/or.2025.9028), which notified the readers that the blots showing β-actin mRNA expression in Fig. 1 on p. 1341 appeared to be similar to the β-actin mRNA blots shown in Fig. 3 on p. 1342, albeit with some horizontal and vertical resizing, another reader has subsequently contacted the Editorial Office to explain that control western blot data featured in the same figures (Figs. 1 and 3) also appeared to be strikingly similar.

We have again reached out to the authors, requesting an explanation for these apparent anomalies in the presentation of the data in this paper; however, up to this time, no response from them has been forthcoming. Owing to the fact that the Editorial Office has been made aware of additional potential issues surrounding the scientific integrity of this paper, we are issuing a second Expression of Concern to notify readers of these additional issues while the Editorial Office continues to investigate this matter.

